# Nutrient intakes and telomere length of cell-free circulating DNA from amniotic fluid: findings from the Mamma & Bambino cohort

**DOI:** 10.1038/s41598-022-15370-9

**Published:** 2022-07-08

**Authors:** Roberta Magnano San Lio, Andrea Maugeri, Maria Clara La Rosa, Giuliana Giunta, Marco Panella, Antonio Cianci, Maria Anna Teresa Caruso, Antonella Agodi, Martina Barchitta

**Affiliations:** 1 Department of Medical and Surgical Sciences and Advanced Technologies “GF Ingrassia”, University of Catania, Via S.Sofia, 87, 95123 Catania, Italy; 2 Obstetrics and Gynecology Unit, Department of General Surgery and Medical Surgical Specialties, University of Catania, Via S.Sofia, 78, 95123 Catania, Italy; 3 Cytogenetic Laboratory, Azienda Ospedaliero Universitaria Policlinico “G.Rodolico - San Marco”, Via S.Sofia, 78, 95123 Catania, Italy

**Keywords:** Biomarkers, Risk factors

## Abstract

Pregnancy represents a crucial period in which several exposures—and especially maternal diet—might shape children’s health. Thus, identifying how maternal dietary intakes early affect biological aging in children represents a public health mission. We aimed to assess the relationship between maternal intake of nutrients in early pregnancy and telomere length of cell-free circulating DNA (cfDNA) from amniotic fluid. We used data and samples from the ongoing prospective “Mamma & Bambino” study, which recruits mother–child pairs from Catania at the first prenatal visit. Maternal nutrient intakes were assessed using a Food Frequency Questionnaire, while relative telomere length of cfDNA was assessed by real-time polymerase chain reaction. Our analysis included 174 mother–child pairs. The intakes of iron, vitamin B1, and magnesium were positively correlated with relative telomere length (p-values < 0.05). However, only the intake of magnesium was positively associated with relative telomere length, after applying a linear regression model (β = 0.002; SE = 0.001; p = 0.024). Magnesium deficiency was negatively associated with relative telomere length after adjusting for the same covariates (β = −0.467; SE = 0.176; p = 0.009). To our knowledge, this is the first evidence of a positive relationship between maternal nutrient intake and telomere length of cfDNA. Further efforts are needed for deeply investigating the effect of maternal dietary intakes on telomere length, in order to develop effective public health strategies.

## Introduction

Human aging is defined as the dynamic process characterized by the recurrent adaptation to internal and external stressors during lifetime^1^, which results in a complex mosaic of the interaction between environmental, genetic and epigenetic events^2,3^. Despite this complexity, several molecular signatures have been proposed to reflect the aging process, also in the context of studying the risk for non-communicable diseases^4^. Among them, the following hallmarks of aging are well investigated: genomic instability^5–8^, telomere attrition^9,10^, epigenetic alterations^11–17^, mitochondrial dysfunction^18,19^, cellular senescence^20–23^, steam cell exhaustion^24^ and altered intercellular communication^25,26^. In vertebrates, telomeres are repetitive sequence of 5′-(TTAGGG)-3′ at the ends of each chromosome, which progressively shorten with cell division giving a partial estimation of the chronological age throughout lifetime^27^. In particular, telomere shortening has been associated with aging^28^ and age-related diseases, such as cardiovascular diseases^29^, cancer^30^, and neurological disorders^31^. The contemporary relationship of telomere length with environmental exposures and lifestyles have made it as an interesting molecular mechanisms in the epidemiological research on age-related diseases^32^.

It is also worth noting that telomere shortening is regulated by epigenetic mechanisms and that telomere length is influenced by DNA methyltransferases and histone methyltransferases^33,34^.

In the last decades, as done for DNA methylation and histone modification^35–38^, several studies have investigated the association of nutrients, foods, and dietary patterns with telomere length. As summarized by Freitas-Simoes and colleagues, the intake of antioxidants and the consumption of plant-derived foods help protect against telomere shortening, while the intake of saturated fats and the consumption of high-sugar and high-calorie products seem to be associated with shorter telomere length^39^. It is also important to note that understanding what maternal factors—and especially maternal dietary habits—might affect biological aging in children could be useful to identify simple strategies for preventing or delaying age-related diseases over the lifetime. In fact, the first 1000 days of life—from conception to two years of age—represents a crucial period in which maternal diet and other exposures might shape children’s health. For instance, some maternal factors, such as stress, smoking and exposure to pollutants, have been associated with shorter telomeres in cord blood^40–42^ and placenta^43^. In addition, a recent systematic review by Habibi and colleagues has sought to unravel how the diet of pregnant women affects telomere length in their offspring^44^. Although some studies reported potential positive (e.g., folate and caffeine) or negative (e.g., fat and sodium) associations with telomere length, the authors stated that the evidence is currently limited and often controversial^44^. Moreover, none of these studies has investigated the effect of maternal dietary factors on telomere length of cell-free circulating DNA (cfDNA) from amniotic fluid^44^. In particular, cfDNA in plasma and serum has been proposed as an innovative biomarker for prenatal diagnosis^45,46^. However, since amniotic fluid contains a greater amount of cfDNA than maternal serum^47–50^, there is a growing interest in investigating the relationship between maternal diet and telomere length of cfDNA from amniotic fluid. To achieve this goal, we used data and samples from the “Mamma & Bambino” cohort to evaluate the relationship between maternal intake of nutrients in early pregnancy and telomere length of cfDNA from amniotic fluid.

## Results

### Characteristics of the study population

The current analysis was conducted on 174 women recruited at a median gestational week of 14 (IQR = 5) and with a median age of 38 years (IQR = 4). Table 1 summarizes the characteristics of the study population. The majority of women (83.3%) reported a high educational level, having a secondary or tertiary education. More than half of them (54.0%), instead, were part-time or full-time employed. The median pre-gestational BMI was 22.8 kg/m^2^ (IQR = 4.6) and thus 21.3% women were overweight or obese before pregnancy. With respect to smoking status, nearly 80% of women were non-current smokers. Table 1 also reports the median intake of nutrients considered in the current analysis. Overall, women had a total energy intake of 1662 kcal (IQR = 634) and their dietary deficiencies varied from 17.8% for vitamin A to 98.3% for iron. About one in three women (35%) took multivitamin or multimineral supplements.

**Table 1:** Characteristics of the study population.

Characteristics (n = 174)	Median (IQR) or frequency (%)
Age, years	38 (4)
High educational level	145 (83.3%)
Employed	94 (54.0%)
Pre-gestational BMI, Kg/m2	22.8 (4.6)
Overweight/Obese	37 (21.3%)
Non smokers	138 (79.8%)
Calories (kcal)	1661 (634)
Vitamin A (IU)	849.0 (557.7)
Vitamin A deficiency	31 (17.8%)
Vitamin B1 (mg)	1.2 (0.6)
Vitamin B1 deficiency	92 (52.9%)
Vitamin B6 (mg)	1.7 (0.6)
Vitamin B6 deficiency	77 (44.3%)
Vitamin C (mg)	106.4 (120.6)
Vitamin C deficiency	52 (29.9%)
Vitamin D (μg)	3.1 (3.8)
Vitamin D deficiency	166 (95.4%)
Folate (μg)	261.9 (142.4)
Folate deficiency	168 (96.6%)
Calcium (mg)	774.6 (377.3)
Calcium deficiency	94 (54.0%)
Iron (mg)	11.1 (6.4)
Iron deficiency	171 (98.3%)
Magnesium (mg)	248.1 (116.5)
Magnesium deficiency	127 (73.0%)
Zinc (mg)	7.6 (2.7)
Zinc deficiency	140 (80.5%)
Monounsaturated fatty acids (g)	34.9 (18.0)
Polyunsaturated fatty acids (g)	10.8 (4.9)
Saturated fatty acids (g)	19.1 (9.7)

### The relationship between relative telomere length and nutrient intakes

**Figure 1:**
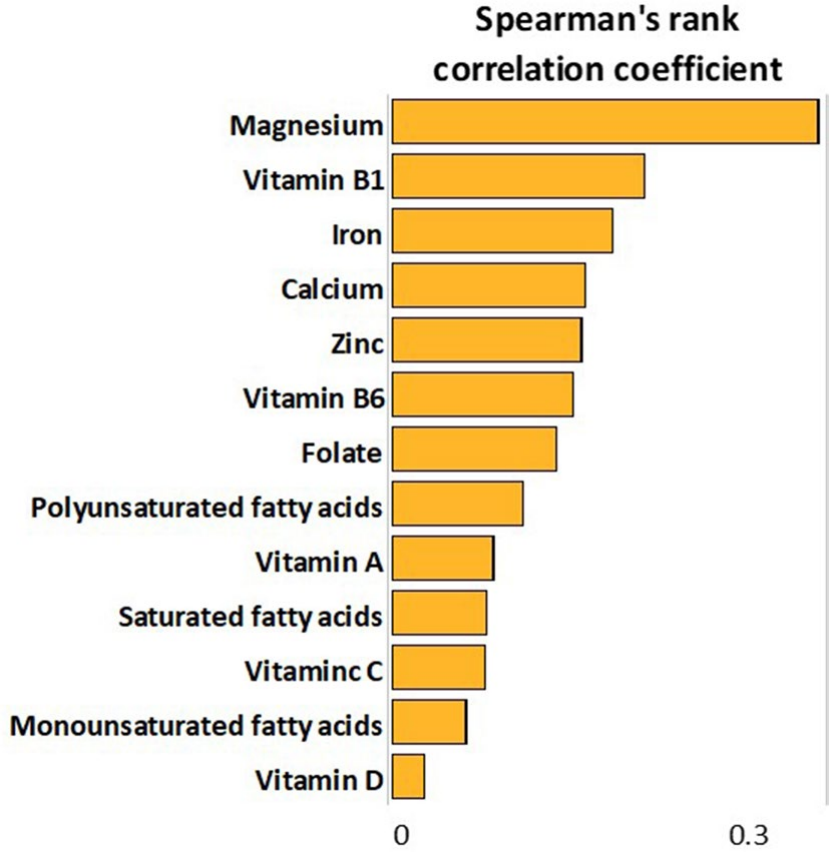
Correlations of nutrient intakes with relative telomere length.

**Figure 2:**
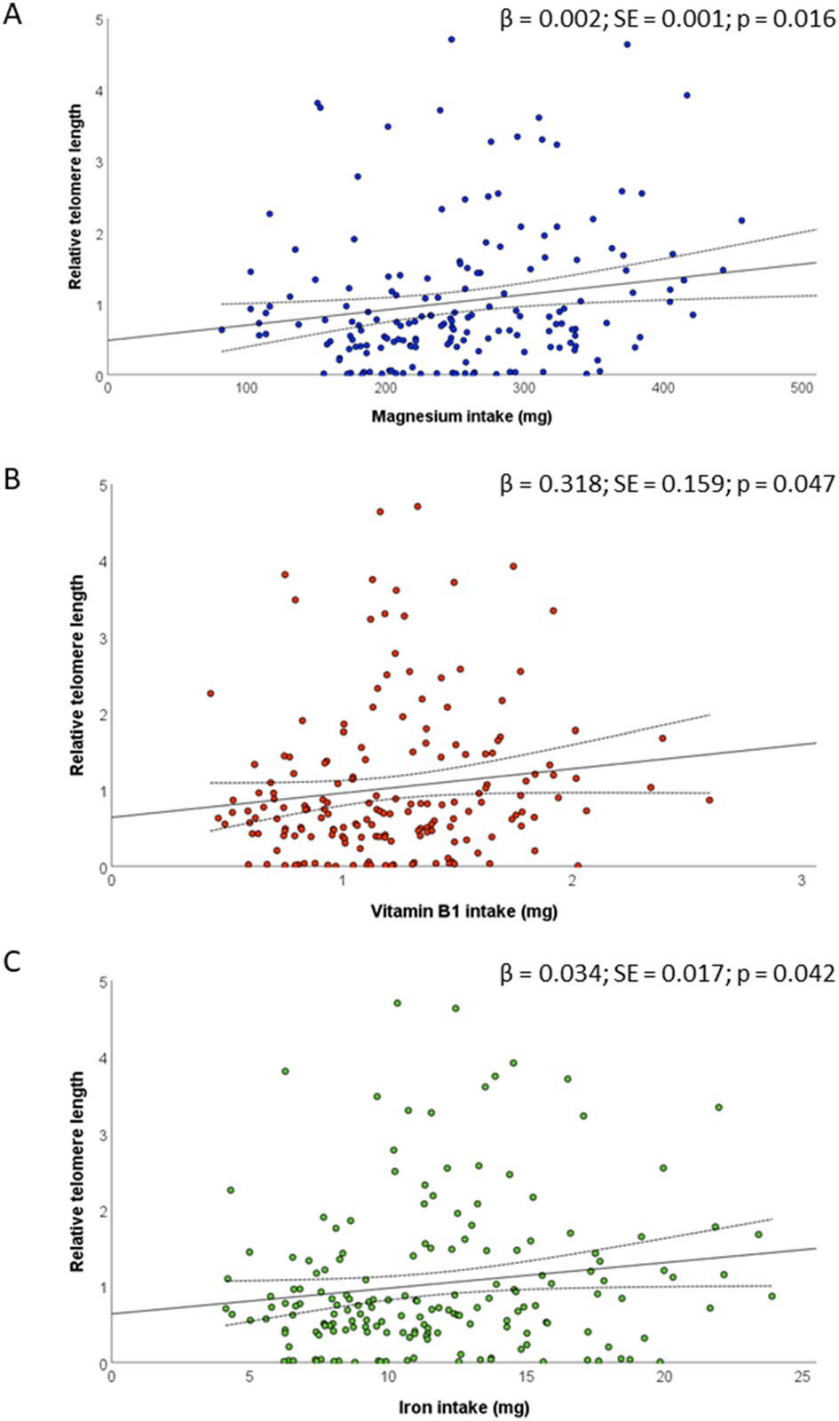
The relationships between relative telomere length and the intake of magnesium (**A**), vitamin B1 (**B**), and iron (**C**). Results are reported as β coefficient and SE.﻿

**Figure 3:**
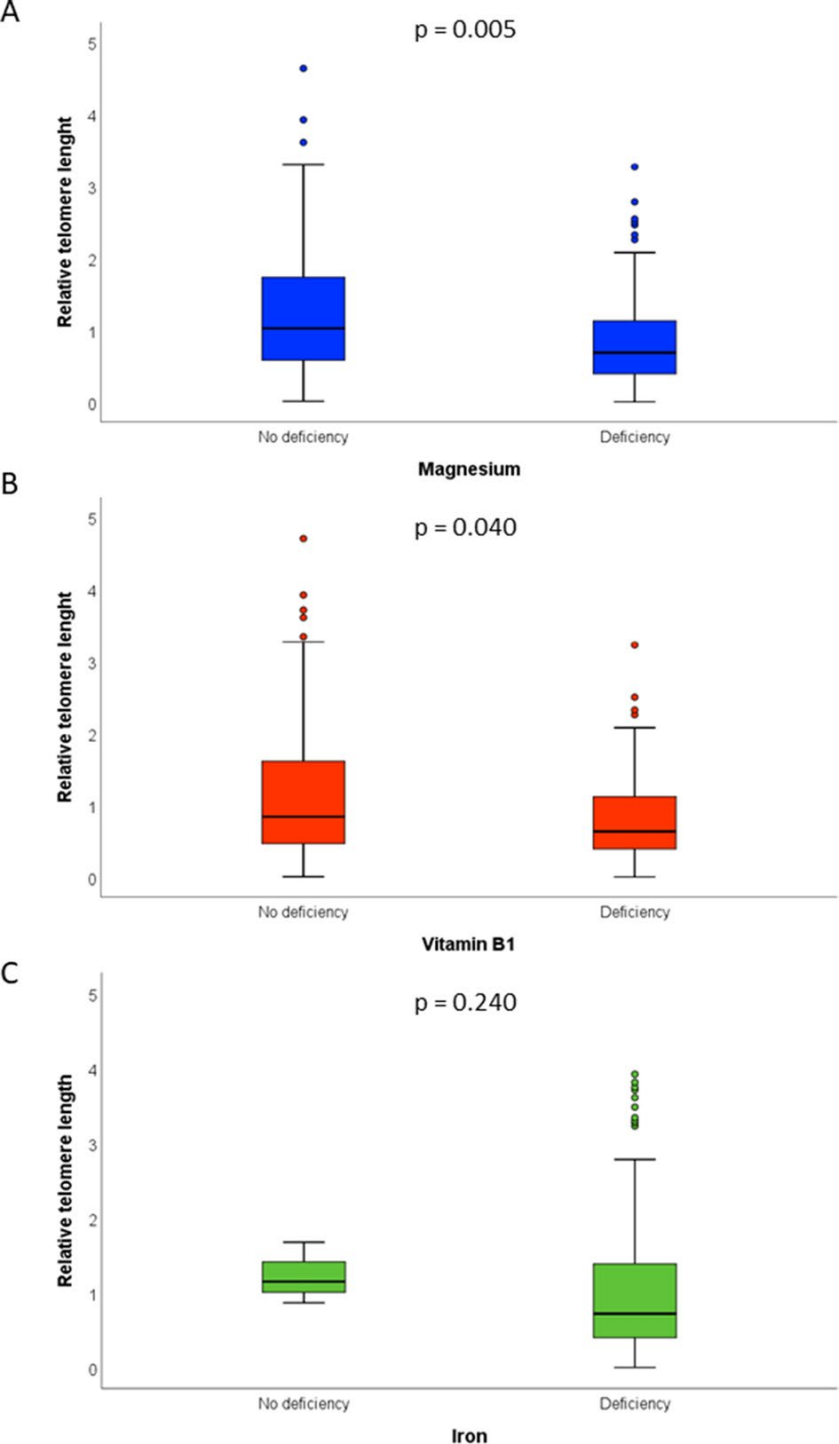
Differences in relative telomere length according to deficiency of magnesium (**A**), vitamin B1 (**B**), and iron (**C**). P-values are based on the Mann Whitney U test.

Overall, median relative telomere length was 0.73 (IQR = 1), while no differences were evident by maternal age, educational level, employment status, pre-gestational BMI, and smoking status (p-values > 0.05). Figure 1 reports the Spearman’s rank correlation coefficients between relative telomere length and nutrient intakes. Most of the relationships were not significant and only the intakes of magnesium, vitamin B1 and iron were positively but weakly correlated with relative telomere length (p-values < 0.05). However, only the correlation with magnesium remained significant after adjusting for multiple comparison. Figure 2 shows positive linear relationships of the intakes of magnesium, vitamin B1, and iron with relative telomere length. However, only the intake of magnesium was positively associated with relative telomere length (β = 0.002; SE = 0.001; p = 0.024), after applying a linear regression model which included maternal age, smoking, pre-gestational BMI, total energy intake, and supplement use. Figure 3 illustrates differences in relative telomere length according to nutrient deficiency. In particular, women with magnesium deficiency (73% of the total population) exhibited lower relative telomere length than those with adequate dietary intake (p = 0.005). Similarly, women with vitamin B1 deficiency (53% of the total population) showed lower values than those with adequate intake (p = 0.040). By contrast, no significant difference was evident according to iron deficiency (p = 0.240). After adjusting for covariates, however, only magnesium deficiency was negatively associated with relative telomere length (β = −0.467; SE = 0.176; p = 0.009).

## Discussion

To our knowledge, this is the first evidence of a relationship between maternal nutrient intake and telomere length of cfDNA from amniotic fluid. In particular, we found positive but weak correlations of intakes of magnesium, vitamin B1 and iron with relative telomere length. While findings on vitamin B1 and iron were not confirmed by further statistical analyses, we demonstrated a positive association between magnesium intake and relative telomere length. The positive effect observed for magnesium remained significant after adjusting for covariates, which hence resulted in shorter telomeres in cfDNA from women with magnesium deficiency. It is worth noting, however, that there is a lack of studies investigating the relationship between magnesium intake and telomere length of cfDNA from amniotic fluid. Although some of our findings were consistent with previous studies, it is important to underline differences in sample types and methods used to perform molecular analyses, which discourage a proper comparison. In vitro and in vivo studies showed that long-term exposure to magnesium deficiency led to telomere shortening^51,52^. In a cross-sectional analysis of the Sister Study, instead, reported a positive association between magnesium intake and telomere length of leukocyte DNA from women who did not use multivitamin supplements^53^. From a biological point of view, magnesium is an important cofactor for the catalytic activity of enzymes implicated in DNA replication and repair^54–57^, and in RNA synthesis^54^. Magnesium deficiency is also often associated with oxidative stress^51^ and pro-inflammatory status^58^, which in turn might lead to telomere shortening.

As discussed above, the evidence remained scarce and inconclusive for other nutrients. In fact, our analysis suggested positive but weak correlations between relative telomere length of cfDNA and the intakes of vitamin B1 and iron. A previous cross-sectional study on Italian subjects failed in demonstrating a relationship between telomere length of leukocyte DNA and the intake of vitamin B1^59^. Moreover, to the best of our knowledge, no studies were conducted during the periconceptional period. Regarding iron, results from the Sister Study did not demonstrate an association between its dietary intake and telomere length^53^, however, it has been suggested a negative effect of iron supplementation^53,60^. Indeed, iron is a prooxidant and its supplementation might increase the production of free radicals^61^, thus fostering an oxidative environment^60^. By contrast, the supplementations with multivitamins, which contained less iron than specific supplements, did not produce a negative effect^53^. The lack of any solid evidence in this field of research, therefore, encourages further efforts to understand the influence of maternal dietary factors on biological aging, as determined by telomere length. Furthermore, future studies investigating other biomarkers of aging could help resolve this question and to translate the answers into effective public health strategies.

The main strength of our study lay on the possibility of investigating the early effect of maternal nutrient intake on biological aging of their offspring, by analysing telomere length of cfDNA from amniotic fluid. In fact, compared to maternal serum, amniotic fluid contains cfDNA largely uncontaminated by maternal-derived nucleic acids. However, as the first study on this topic, our results should be confirmed by further analyses. Our work had also some limitations that should be considered when interpreting our results. Firstly, the limited sample size did not allow us to perform additional analyses for residual confounders. However, as showed by Habibi and colleagues, our study was, at the time of writing, one of the larger in its field. Secondly, the FFQ that we used for dietary assessment—although simple, time- and cost-efficient—did not preclude subjective assessment, measurement errors and inaccuracies^62^. Moreover, this tool did not consider any changes related to food cooking. To partially address these issues, the use of some biomarkers of validation (e.g., serum level of nutrients) could have made the evidence more solid. Thirdly, differences in analysed samples, methods, and kits used for DNA extraction and telomere length assessment did not allow a proper comparison with previous studies. Although amniotic fluid is considered a relatively pure fetal sample, a low proportion of cfDNA from placenta cannot be completely excluded^63^. Indeed, placenta could contribute to cfDNA in amniotic fluid, especially in women with placental abnormalities and preeclampsia. Accordingly, we excluded from our analysis all women with pregnancy complications. cfDNA is also inherently degraded and therefore DNA strands are highly fragmented if compared with cellular DNA from other samples. Moreover, the extraction kit used in our study was not specifically developed for cfDNA from amniotic fluid, even if it was appropriate for body fluids in general. Regarding telomere length assessment, the qPCR used in our study had higher assay variability than terminal restriction fragment analysis used by previous studies^64^. For these reasons, further validation analyses and comparative studies—using different samples, as well as alternative methods for DNA extraction and telomere length assessment—should be encouraged.

Finally, although we adjusted the analysis for some factors that could influence the observed relationship, we cannot completely exclude the effect of unmeasured confounders.

In conclusion, we found a positive association between maternal intake of magnesium and telomere length of cfDNA from amniotic fluid, while results on other micronutrients (i.e., vitamin B1 and iron) were marginally significant. This is the first evidence of an early effect of maternal magnesium intake on biological aging of offspring. Although our approach could help to understand molecular mechanisms underpinning the transgenerational effects of maternal diet on biological aging, further research is needed to identify strategies for preventing or delaying age-related diseases as early as pregnancy.

## Methods

### Study design

The “Mamma & Bambino” cohort is a prospective study that recruits pregnant women during the prenatal genetic counseling (at 4th–20th gestational week) with planned follow-up of their children at delivery and up to two years of age. Full details of the study design and procedures are described elsewhere^65–70^. In brief, the recruitment of women referring to the Azienda Ospedaliero Universitaria Policlinico “G. Rodolico—San Marco” (Catania, Italy) started in 2015 and the study is still ongoing. Mothers with multiple pregnancy, pre-existing autoimmune and/or chronic diseases, and complications, such as preeclampsia, gestational hypertension and diabetes, intrauterine fetal death, and congenital malformations, are excluded. Accordingly, the current analysis was conducted on 174 women (aged 15 to 48 years), who satisfied inclusion criteria reported above. The study is conducted in accordance with the Declaration of Helsinki and its protocol was approved by Ethics Committee of the “Azienda Ospedaliero Universitaria Policlinico-Vittorio Emanuele” and by the Ethics Committee “Catania 1” with the protocol numbers 47/2014/VE, 48/2015/EMPO, 186/2015/EMPO, 197/2016/EMPO, 213/2017/EMPO, 231/2018/EMPO, and 263/2019/EMPO. All participants or their legal guardian give a written informed consent to participate in the study. For the current analysis, we used data and samples from women who completed pregnancy and who provided an aliquot of amniotic fluid obtained through amniocentesis^71^.

### Data collection

For each woman, information on socio-demographic characteristics and lifestyles are collected through structured questionnaires administered by trained epidemiologists. Full details on data collection and management are reported elsewhere^65–71^. In particular, dietary data are collected through a 95-item semiquantitative Food Frequency Questionnaire (FFQ) referred to one month before recruitment, as reported by previous studies on Sicilian women^66,67,72–78^. For each item, information on frequency of consumption and portions size are collected to calculate their daily dietary intake. Next, the intakes of calories, minerals (iron, calcium, magnesium, and zinc), fatty acids (saturated, monounsaturated, and polyunsaturated), and vitamins (A, B1, B6, C, D, and folate) are computed using the U.S. Department of Agriculture (USDA) Food Composition Database (http://ndb.nal.usda.gov/) adapted to typical Italian foods. Nutrient intakes are considered as continuous values or categorized according to the Recommended Dietary Allowance by the Food and Nutrition Board of the Institute of Medicine^79^. At recruitment, each woman reported her height and weight before pregnancy to compute the pre-pregnancy BMI, and hence all women were classified as underweight, normal weight, overweight or obese according to WHO criteria^80^.

### DNA extraction and relative telomere length assessment

Different samples types—including maternal blood, amniotic fluid, cord blood and placenta—are collected from mother–child pairs included in the Mamma & Bambino cohort. In the current analysis, we used an aliquot of 1 ml amniotic fluid obtained from women who underwent amniocentesis, as previously reported^71^. In brief, after centrifugation at 12,500 g, the cfDNA was extracted using the QIAamp Blood Kit (Qiagen, Milan Italy), which is suitable to extract DNA from blood, plasma and serum, cultured cells, swabs, and body fluids. The entire procedure was automatically performed on the QIAcube instrument (Qiagen, Milan, Italy). Quantity and quality of cfDNA were assessed using the dsDNA HS Assay Kit (Thermo Fisher Scientific, Carlsbad, CA, USA) on the Qubit 3.0 Fluorometer and the NanoDrop 1000 spectrometer. Next, relative telomere length of cfDNA was evaluated real-time quantitative polymerase chain reaction (qPCR), using the Relative Human Telomere Length Quantification Assay Kit (ScienCell Research Laboratories, Carlsbad, CA, USA) on the QuantStudio 7 Flex Real-Time PCR System (Thermo Fisher Scientific, Carlsbad, CA, USA). Full details on the qPCR protocol are described elsewhere^81^. All reactions were run in duplicate and relative telomere length was expressed as telomere/single copy reference (T/S) ratio. The procedures described above were conducted according to the manufacturers' protocols, unless otherwise stated.

### Statistical analysis

The characteristics of the study population were described using frequencies (percentage, %) for qualitative variables, and using median and interquartile range (IQR) due to the skewness of quantitative variables. The correlations between nutrient intakes and relative telomere length were tested using the Spearman's rank correlation test and adjusting for multiple comparisons with Bonferroni correction. For nutrients that showed a significant correlation (p < 0.05), we plotted their continuous value against relative telomere length, and then we compared relative telomere length between deficient and not deficient women using the Mann Whitney U test. Finally, we tested the association between nutrient intake (as continuous or categorical variable) and relative telomere length, adjusting for factors that could influence nutrient intake and/or telomere length (i.e., maternal age, smoking status, pregestational BMI, total daily energy intake, and supplement use). The adjusted association was reported as β coefficient and its standard error (SE). All the statistical analyses were performed using SPSS (version 26), all tests were two-sided and performed at a significance level α = 0.05.

### Institutional review board statement

The study was conducted according to the guidelines of the Declaration of Helsinki, and approved by the Ethics Committee of Azienda Ospedaliero-Universitaria Policlinico-Vittorio Emanuele” and Ethics Committee “Catania 1″ with the following protocol numbers: 47/2014/VE; 48/2015/EMPO; 186/2015/EMPO; 197/2016/EMPO; 213/2017/EMPO; 231/2018/EMPO; 263/2019/EMPO.

### Informed consent

Informed consent was obtained from all subjects involved in the study.

## Data Availability

The datasets analyzed during the current study are available from the corresponding author on reasonable request.
